# Sir Hugh Cairns: a pioneering collaborator

**DOI:** 10.1007/s00701-019-03934-0

**Published:** 2019-05-08

**Authors:** Jonathan Edward Attwood, Gabriele C. De Luca, Terence Hope, Deva Sanjeeva Jeyaretna

**Affiliations:** 1 Nuffield Department of Clinical Neurosciences, Level 6 West Wing, John Radcliffe Hospital, Headley Way, Oxford, Oxfordshire OX3 9DU UK; 2 Department of Neurosurgery, Nottingham, Nottinghamshire UK; 3 Department of Neurosurgery, John Radcliffe Hospital, Oxford, UK

**Keywords:** Sir Hugh Cairns, History of neurosurgery, Military neurosurgery

## Abstract

In April 1988, Peter Schurr delivered the twelfth Sir Hugh Cairns Memorial Lecture to the Society of British Neurological Surgeons. In his lecture, *The Cairns Tradition*, Schurr extolled the personal virtues of Cairns. He encouraged his colleagues to draw inspiration from Cairns’ renowned determination, organisation, drive for perfection, compassion, and commitment to the training of those around him. Indeed, Cairns’ own personality has come to define the specialty which he established in Britain. Today’s neurosurgeons are, whether knowingly or not, formed in his image. But there is a side to Hugh Cairns that has been lost in the telling of his remarkable story, and yet it played a central role in his greatest achievements. This is the side of himself which he turned towards others. Throughout his career, Cairns received an inordinate number of personal accolades. His tutelage under Cushing during a formative trip to America and the impact of his role in caring for T. E. Lawrence are well known to many. But, more than thirty years after Peter Schurr’s memorial lecture, and following the eightieth anniversary of the department of neurosurgery founded by Cairns in Oxford, it is his work as a pioneering collaborator which defines his legacy today, and which calls us to learn yet another lesson from his remarkable life. In this legacy article, we review the origins of Cairns’ collaborative spirit and uncover the achievements he shared with Charles Hallpike, Howard Florey, Derek Denny-Brown, William Ritchie Russell, Ludwig Guttman, and Peter Medawar, among many others.

## Introduction

Sir Hugh Cairns was a pioneering collaborator. From the days of his youth spent on the sports field and on stage, to his time as a medical student serving in the First World War, the foundations were laid for a career sustained by co-operation. Cairns has historically been portrayed as stern, demanding, and somewhat inpatient by colleagues who knew him well. Indeed, he may not have been overly forthcoming in all his dealings with others. For example, he deliberated for six months before sharing his surgical specimens with the otologist Charles Hallpike. But Cairns chose his colleagues well, and his work with Hallpike leads to the first description of the pathophysiology of Ménière’s disease. Cairns’ collaboration with pathologist Howard Florey, by contrast, was built on a lifelong friendship, and, it seems, was even more successful as a result. With Florey, who carried out the first clinical trial of penicillin in 1941, Cairns not only helped to establish the clinical medical school at Oxford University, but also pioneered the use of antibiotics in surgery during the Second World War.

Yet perhaps the best example of Cairns’ collaborative spirit was the formation of the Military Hospital for Head Injuries at St Hugh’s College in Oxford. In the midst of war, and under the roof of a converted undergraduate college, Cairns brought together the brightest physicians, surgeons, and scientists in Britain. This extraordinary team treated more than 13,000 servicemen and women, but, what is more, made discoveries that have shaped our understanding of concussion and traumatic brain injury, established the field of transplant surgery, and led a revolution in the understanding of disability and rehabilitation.

### Origins of a collaborator

Hugh Cairns was born and raised on the south coast of Australia at the turn of the twentieth century, the only son of William and Florence Cairns. He won a bursary to Adelaide High School in 1908, where he played tennis and football, and was described by the headmaster as “a bright, frank, and candid boy—always popular but never seeking to be…always top of his class and finally top of the school” [1]. He went on to earn an Exhibition to Adelaide University, and began to study medicine in 1912, at the age of fifteen. Cairns would later recall his memories from the time, “in the dissecting room, Watson would give us a talk on some anatomical subject, and would embellish it by graphics, and sometimes enliven it with descriptions of operations that had failed because this or that anatomical point had been ignored. Then we would adjourn to ride the Professor’s motor bike around the dissecting room while he timed us with a stop-watch”.

With certainly with no shortage of entertainment to accompany his studies, Cairns continued to achieve highly, obtaining first-class honours in his first and third year examinations, while also finding the time to row, play lacrosse, football, and tennis, and compete in athletics for the university. In his second year, perhaps at the expense of his exams, he played a female role in the Varsity Rag, starring opposite his anatomy classmate Alan Morey. Cairns went on to follow in Morey’s footsteps by winning the Rhodes Scholarship to study in Oxford three years after his classmate. But, as it happened, the studies of both young men were interrupted by the outbreak of war in Europe.

### Military service in the First World War

Cairns joined the Australian Army Medical Corps as a Private in 1914, and served in support of the Gallipoli landing in April the following year. Aged nineteen, midway through his fourth year at medical school, he travelled to the Middle East via Columbo with the 3rd Australian General Hospital. Still a young man, his mother wrote to Hugh imploring him to sleep on deck rather than in the hold, “on the condition that he must masquerade as a woman in the event of an attack by a raider” [1]. He passed through London for the first time in June, before arriving at the island of Lemnos in July, where his unit set up to provide medical support to the Gallipoli peninsula.

They arrived to find a “bare and treeless hillside, without tents or equipment, without water-supply except tank-ships and one water-cart, and without sanitary provision…they bivouacked among flat rocks without any shelter” [1]. The unit constructed bell-tents to serve as operating theatres, equipped with mattresses but no beds, nurses, orderlies, radiology, or pathology. The conditions were harsh, but Cairns was dedicated, and soon found himself “in full charge of the x-ray department…and was said to show the greatest keenness and enthusiasm for this work, a cheerful disposition, and a ready willingness to do whatever work presented itself” [1]. He was promoted to acting Lance-Corporal, and before long his unit supported over a thousand medical beds, treating sick and wounded soldiers returning from the north African theatre, and acting as a vital staging post on the route back to England.

The field hospital on Lemnos was “a social centre for all ranks, as well as a Mecca to regimental medical officers hungry for…clinical discussion and scientific surrounding” [1]. Cairns recorded in his own diary: “…saw good direct inguinal hernia…went to bed early and read Surgeon’s Log” [1]. His education in the field appears to have been formative, but it came to an end in February 1916, when medical students were instructed to return home to complete their courses. Cairns passed the year with first class honours, and as the war continued, he was called upon again to perform the duties of the hospital pathologist, and once more rose to the occasion, doing so “to the complete satisfaction of all concerned” [1].

The war brought bitter challenges and tragic losses to all, but in the face of adversity, Cairns was able to thrive. He rose through military ranks and found his place working with and for others, as well as realising lifelong interests in surgery, radiology, and pathology. He passed his final examinations in June 1917, still the youngest in his class, and by the time the war had ended, he won the South Australian Rhodes Scholarship to continue his studies in Oxford.

### Oxford years

During Cairns’ time in Oxford, he was fortunate to study under the auspices of some of the greatest physicians and scientists of the Victorian era. His physiology class was given by Sir Charles Sherrington, whose work on synapses, a term which he himself coined, would later receive the Nobel Prize. He was taught anatomy by Sir Wilfrid Le Gros Clark, after which the main building of the Department of Physiology, Anatomy and Genetics at Oxford is named, and he dutifully attended Sir William Osler’s Sunday morning teaching rounds at the Radcliffe Infirmary. Cairns continued to work hard as a graduate student and gained an excellent reputation. Before long, he found himself invited to dinner with the Sherringtons, and, on one occasion, was even called upon to pull Mrs. Sherrington out of the river Thames, after she had fallen in from the Professor’s punt.

In the years after the war, it was on the water where Cairns continued to excel as a teammate and increasingly as a leader. He rowed in the 1920 Oxford and Cambridge Boat Race, and went on to coach the crew of Balliol College at the Henley Regatta. For this particular endeavour, Cairns sought the help of none other than Rudyard Kipling, who, after meeting the crew at dinner in college, was convinced by Cairns to come out to the river, time the boat, and shout encouragement from the launch. As a colleague later remarked, “Hugh was always one to enlist advice and to get the right people, people with different knowledge and different influences, people who could help, on his side” [7].

### Cairns and Hallpike: the pathophysiology of Ménière’s disease

Cairns left Oxford to complete his surgical training in London. He became the first full-time neurosurgeon in Britain, and during this time, he came across two patients who were suffering with the disabling vertigo, tinnitus, and hearing loss of Ménière’s syndrome, and who required the delicate operation of an eighth cranial nerve division. Sadly, though not uncommonly at the time, both patients died from postoperative intracerebellar haemorrhage. Characteristic of Cairns’ humility, and following his interest in pathology, he preserved the temporal bones from these cases, and “after six months’ thought and enquiry as to whose would be the safest hands to receive the precious material” [7], he reluctantly gave them to the otologist Charles Hallpike at Middlesex Hospital. Hallpike later told Sir Geoffrey Jefferson “how very carefully he was cross-examined and how cagely [sic] he was looked over before Cairns thought it safe to put the bones into his hands for histology, with what eventual result everybody knows” [7]. In May 1938, after identifying endolymphatic hydrops in the bones of both patients, Cairns and Hallpike presented their *Observations on the Pathology of Ménière’s Syndrome* to the Royal Society of Medicine [4]. With a growing reputation both as a surgeon and an academic, Cairns returned to Oxford to take up the role of the first Nuffield Professor of Surgery, and in this capacity, he immediately set about establishing a neurosurgical department at the Radcliffe Infirmary, which was completed in 1938.

### Cairns and Florey: the Oxford clinical school

Like Hugh Cairns, Howard Florey was also raised in South Australia by parents who had emigrated from Britain. Florey, too, studied medicine at the University of Adelaide and came to Oxford as a Rhodes scholar four years after Cairns. By 1935, Florey had completed his PhD in Cambridge and was made Professor of Pathology at Oxford. Cairns wrote to his old friend one night, “I am very anxious to have a talk with you…it sounds frightfully important, doesn’t it, but I don’t know whether you will think so. It is just an idea about clinical medicine in Oxford” [1]. Cairns’ idea, inspired by his time at Harvard Medical School with Harvey Cushing, was for pre-clinical graduates to receive three years of postgraduate clinical training in return for assisting with medical research being performed by the “laboratories in the Parks”. His hope was that by combining clinical training with scientific research, a clinical school in Oxford would be well placed to train the specialists needed to take up posts in the growing fields of tertiary care which were expanding in Britain at the time.

With Florey’s guidance and encouragement, Cairns met with the professors of the physiology, anatomy, pathology, biochemistry, and pharmacology laboratories to explain his plans to them individually, before coordinating the makings of a new faculty. He drew up the finances of the clinical school himself, and through an impassioned speech to the car manufacturer and philanthropist Lord Nuffield, benefaction was secured. Cairns later recalled, “I talked for an hour, with Lord N. saying practically nothing—I didn’t give him much chance to” [1]. As Florey later wrote, “you can take it that there is no question whatever that it was Cairns’ initiative that started Nuffield on the idea of founding a substantial addition to the medical school here”.

The clinical school in Oxford was established as a collaboration between the professors of pre-clinical science and the hospital physicians, and in this way, it reflected the close relationship between Florey and Cairns. Between 1936 and 1938, Cairns wrote to Florey every four or five days. They were a good match for each other, as one colleague recalled, “Florey was as stubborn and indomitable as was Hugh himself” [7], and Cairns’ diaries describe countless dinners in candle-light college halls with Florey, Le Gros Clark, and others throughout 1938, as they expanded their plans for the undergraduate course.

### The military hospital for head injuries

**Fig. 1:**
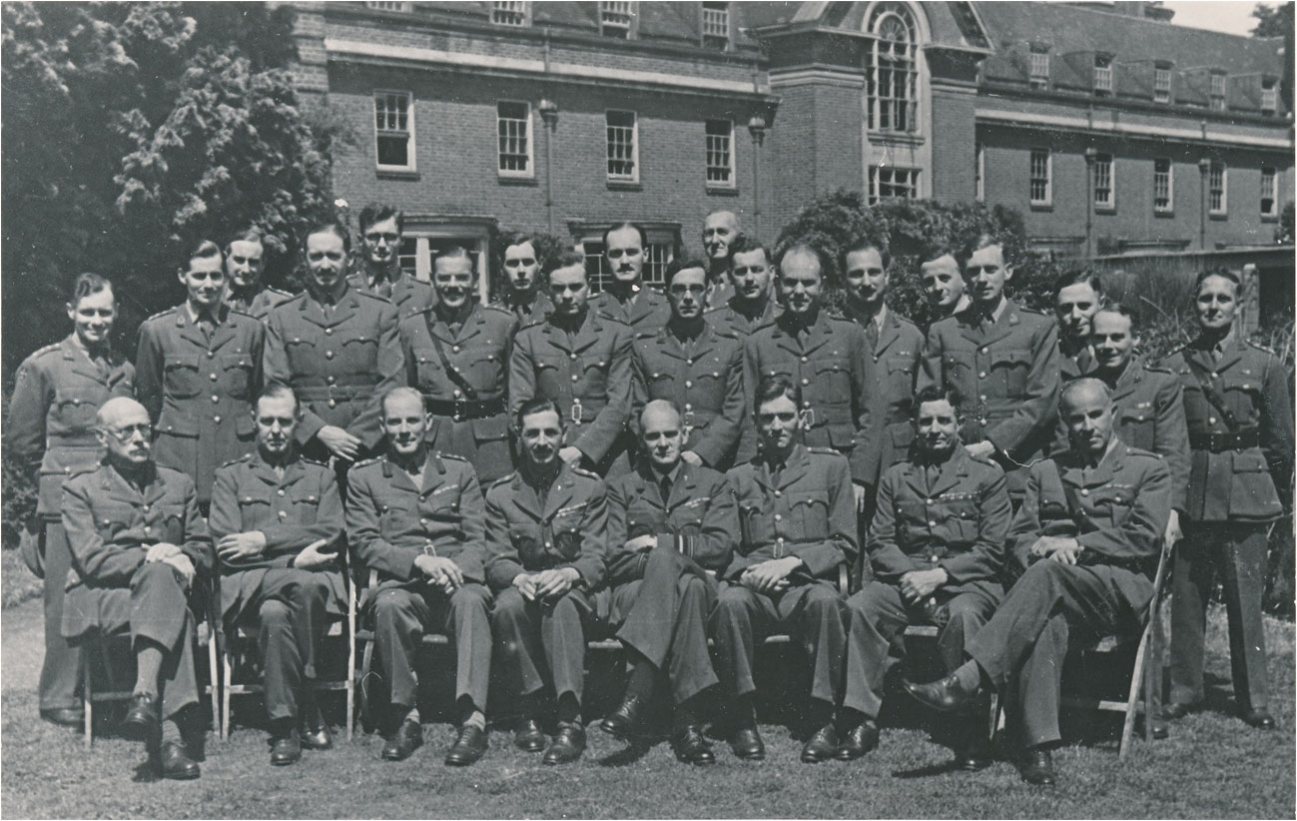
The staff of the Military Hospital for Head Injuries, including Sir Hugh Cairns (seated third from left), Sir Charles Symonds (seated fourth from right), and William Ritchie Russell (seated third from right). Reproduced by kind permission of the Principal and Fellows of St Hugh’s College, Oxford

**Fig. 2:**
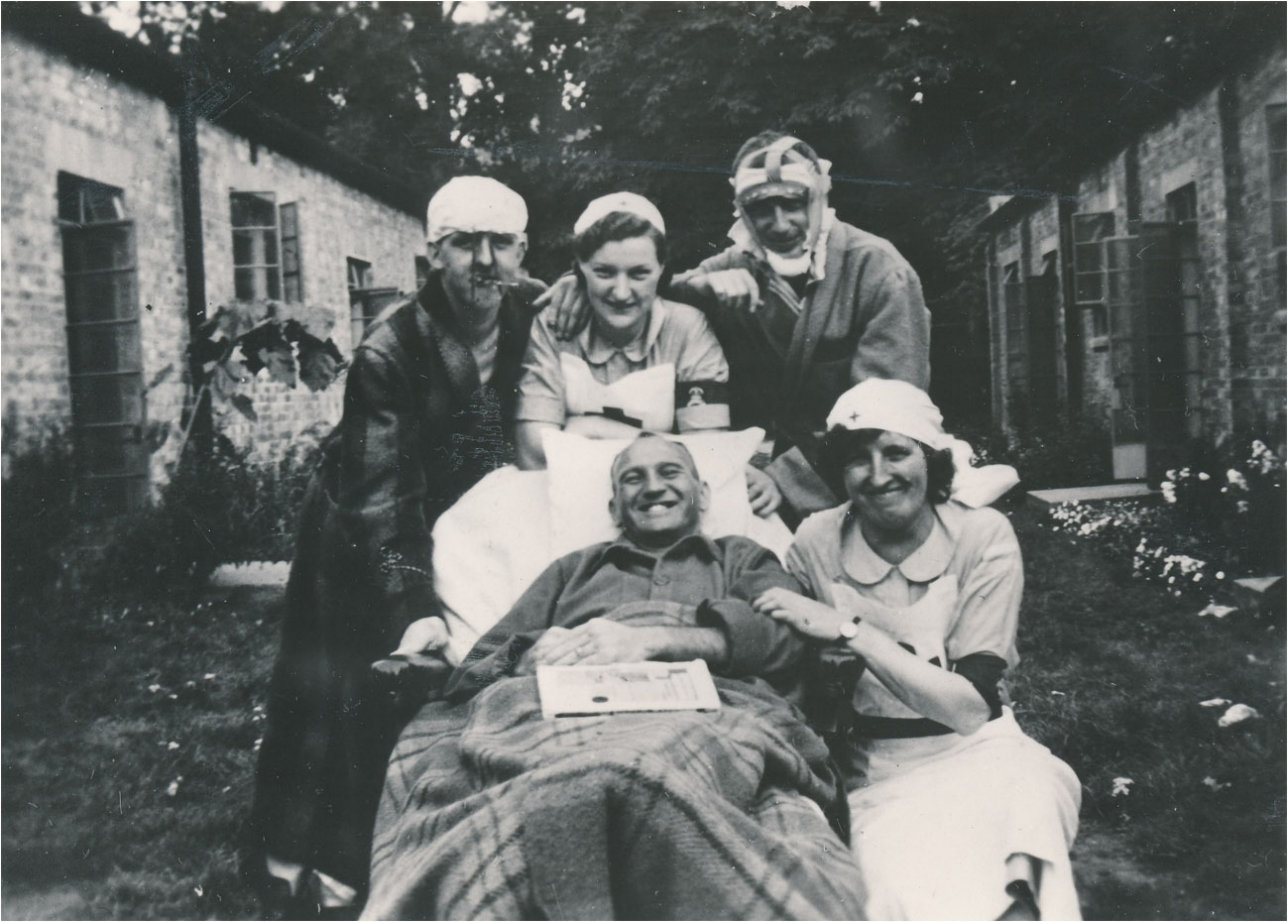
Patients and nurses at the Military Hospital for Head Injuries. Reproduced by kind permission of the Principal and Fellows of St Hugh’s College, Oxford

Cairns and Florey’s work on the fledgling medical school was short-lived. As peace in Europe came under threat once more, Cairns was called upon by the War Office to make preparations for the many injured soldiers expected to return from France. This was to be by far the most intense period of collaboration for Cairns, as he was charged with forming a national Military Hospital for Head Injuries, which he established at St Hugh’s College Oxford in 1940 (Figs. 1 and 2).

Sir Charles Symonds, consultant neurologist for the Royal Air Force and Guy’s Hospital in London, was appointed to lead the neurology department at St Hugh’s. It was clear from the outset, however, that Cairns was in charge. As Symonds later recalled, “at St Hugh’s he and I got on happily together, though there were one or two collisions and sore heads before I learned that I served in Cairns’ hospital” [10]. Cairns and Symonds joined the Committee on Brain Injuries formed by the Medical Research Council in 1940, and through the study of the wounds sustained by British soldiers returning from Europe to their care, they produced ground-breaking findings on the nature of concussion and the role of intracranial pressure and brain swelling in penetrating head injury.

A fine team of young neurologists worked alongside Cairns and Symonds in the care of soldiers at St Hugh’s, including Derek Denny-Brown and William Ritchie Russell, who would go on to become professors of neurology at Harvard and Oxford respectively after the war. Cairns insisted that every history, examination, investigation, and operation be performed systematically and documented meticulously. Amid the chaos of war, he instilled his own precision and clarity in those he worked with, so that St Hugh’s became not only an efficient military hospital and a sanctuary for those returning from war, but also a frontier of medical discovery in its own right.

Together, Cairns, Denny-Brown, and Russell took leading roles in the study of post-traumatic amnesia and epilepsy, and the rehabilitation of soldiers with head injuries. Russell, in particular, recognised the men treated at St Hugh’s as both a unique medical cohort and a group who embodied the sacrifices made by so many for their country, which like them would survive the war, but would have to learn how to live with the wounds it left behind. Russell cared for and assessed the St Hugh’s soldiers for the rest of his career, documenting not only the remarkable capacity of the nervous system to recover from traumatic injuries over decades, but also the impact of disability on their modern lives [8]. He was joined in this work by Ludwig Guttmann, a young Jewish surgeon who escaped Nazi Germany in 1939, and who was brought to Oxford with his wife and two children by the Council for Assisting Refugee Academics. Under Cairns’ tutelage at St Hugh’s, Guttmann established the role of surgery and physiotherapy for patients with peripheral nerve injury [2, 3], and went on to establish the National Spinal Injuries Centre at Stoke Mandeville Hospital in Buckinghamshire in 1943. In 1948, at the same time as the Olympic Games were taking place in London, Guttmann decided to organise a parallel games for veterans with spinal cord injuries. Guttman’s games resonated with the public mood in a year that also saw the formation of the National Health Service. They were deemed a major success, and have grown into what we now know as the Paralympic Games.

Throughout the war, Cairns also continued to work closely with Florey. Florey and Chain had recently isolated penicillin after Alexander Fleming’s discovery of the antibacterial mould in 1928. Florey informed the War Office of their progress, and in 1943, he and Cairns were instructed to trial the treatment in North Africa. Their trip would serve to transform the face of modern medicine, as the childhood friends became the first to demonstrate the life-saving potential of antimicrobials on a meaningful scale. They administered penicillin as both prophylaxis and treatment of cranial infections and cases of pyogenic meningitis. Their preparation of penicillin was less than 50% pure, and they were aware that it did not readily cross the blood-brain barrier. But the direct application of penicillin powder to cranial wounds following neurosurgical debridement lowered the rate of postoperative infection from 4.4% to 0.9%. They also administered intrathecal penicillin and devised a means of achieving therapeutic doses by measuring levels in the cerebrospinal fluid, and, as a result of their work, records show that by 1945, British military casualties due to brain abscess had fallen from 27% to just 3% [9].

And yet, somehow, this was by no means the last of Cairns’ collaborations at St Hugh’s. He also brought together and worked with the eminent immunologist Peter Medawar and biologist John Zachary Young, whose work in nerve grafting and regeneration established the immunological principles which underpin the modern field of transplant surgery. In addition, Cairns also found time to work with the young physicist A.H.S Holbourn, who analysed the physical properties of the brain and skull and the mechanics of head injury to establish the central role of acceleration and shear strain in producing brain damage and contrecoup injuries [5]. Cairns and Holbourn analysed over 100 cases of motorcycle head injuries and showed that wearing a pulp crash helmet reduced the incidence of skull fractures by 75%, hospital admission by 50%, and amnesia by one-third following head injury [6]. Crash helmets immediately became compulsory for motorcyclists in the military, and were soon adopted by the police before being made a legal requirement for all motorcyclists in the UK.

## Conclusion

At St Hugh’s College during the Second World War, Cairns assembled a team that became worth far more than the sum of its parts. Long after the war had ended, the relationships formed and the insights gained there continued to flourish, and have served to define many central aspects of modern neurology, neurosurgery, trauma, immunology, and rehabilitation medicine. Cairns himself continued to operate at the Radcliffe Infirmary in Oxford and collaborated with physicians, scientists, and increasingly psychiatrists until his untimely death on 18 July 1952.

Cairns seems to have thrived alongside colleagues from such a variety of disciplines because he was able to recognise a part of himself in those with whom he worked. He was trained as both a physician and a surgeon, an anatomist and a physiologist, held lifelong interests in pathology and radiology, and saw himself as both a student and a teacher. In his own words, “nearly every young doctor should try his hand at…investigation—should try to carry knowledge of some disease or symptom or function, or the effect of some form of treatment a stage further than it has been carried before. Otherwise he will not find out what he is good for, nor will he appreciate the difficulties and labours involved in discovering new things” [9]. His intellectual adaptability and seemingly limitless energy in the pursuit of medical progress were embodied not only in successful endeavours in research, but in the creation of a unique hospital which served people at a time of crisis while pushing the boundaries of scientific progress, and in the founding of a clinical school which holds his personal ideals at its heart to this day.

Sir Hugh Cairns is often remembered as the man who established neurosurgery in Britain. But it is perhaps more important to remember how he was able to achieve this feat, and in so doing commit himself fully to the highest quality of care for his patients: by sharing his time, energy, and resources with colleagues from every discipline.
